# Temperature alters reproductive life history patterns in *Batrachochytrium dendrobatidis*, a lethal pathogen associated with the global loss of amphibians

**DOI:** 10.1002/ece3.334

**Published:** 2012-08-04

**Authors:** Jamie Voyles, Leah R Johnson, Cheryl J Briggs, Scott D Cashins, Ross A Alford, Lee Berger, Lee F Skerratt, Rick Speare, Erica Bree Rosenblum

**Affiliations:** 1 Department of Environmental Science, Policy and Management, University of California- BerkeleyBerkeley, California, 94720-3144, USA; 2 Department of Ecology and Evolution, University of Chicago1101 E. 57th Street, Chicago, IL, 60637, USA; 3 Department of Ecology, Evolution and Marine Biology, University of CaliforniaSanta Barbara, CA, 93106, USA; 4 School of Public Health, Tropical Medicine and Rehabilitation Sciences, Amphibian Disease Ecology Group, James Cook UniversityTownsville, Queensland, 4811, Australia; 5 School of Marine and Tropical Biology, Amphibian Disease Ecology Group, James Cook UniversityTownsville, Queensland, 4811, Australia

**Keywords:** Amphibian declines, *Batrachochytrium dendrobatidis*, chytridiomycosis, climate change, emerging infectious disease, temperature

## Abstract

Understanding how pathogens respond to changing environmental conditions is a central challenge in disease ecology. The environmentally sensitive fungal pathogen *Batrachochytrium dendrobatidis* (*Bd*), which causes the amphibian disease chytridiomycosis, has spread globally causing amphibian extirpations in a wide variety of climatic regions. To gain an in-depth understanding of *Bd*'s responses to temperature, we used an integrative approach, combining empirical laboratory experiments with mathematical modeling. First, we selected a single *Bd* isolate and serially propagated two lineages of the isolate for multiple generations in two stable thermal conditions: 4°C (cold-adapted lineage) and 23°C (warm-adapted lineage). We quantified the production of infectious zoospores (fecundity), the timing of zoospore release, and zoospore activity in reciprocal temperature transplant experiments in which both *Bd* lineages were grown in either high or low temperature conditions. We then developed population growth models for the *Bd* lineages under each set of temperature conditions. We found that *Bd* had lower population growth rates, but longer periods of zoospore activity in the low temperature treatment (4°C) compared to the high temperature treatment (23°C). This effect was more pronounced in *Bd* lineages that were propagated in the low temperature treatment (4°C), suggesting a shift in *Bd*'s response to low temperature conditions. Our results provide novel insights into the mechanisms by which *Bd* can thrive in a wide variety of temperature conditions, potentially altering the dynamics of chytridiomycosis and thus, the propensity for *Bd* to cause amphibian population collapse. We also suggest that the adaptive responses of *Bd* to thermal conditions warrant further investigation, especially in the face of global climate change.

## Introduction

In many disease systems, the manifestations of disease (e.g., morbidity and mortality) exhibit patterns that can be attributed to changes in environmental factors, such as temperature (Altizer et al. 2006). Especially in systems where host organisms are ectothermic, temperature can have dramatic effects on both the host (e.g., by mediating immune responses) and the pathogen (e.g., by altering reproductive rates). Understanding pathogen responses to changing thermal conditions, decoupled from host defensive responses, is challenging, but important to understanding disease dynamics. This is particularly true in disease systems where environmental temperatures have strong effects on pathogen abundance, distribution, and life history patterns (Harvell et al. 2002; Lafferty 2009). One such disease is chytridiomycosis, caused by the fungal pathogen *Batrachochytrium dendrobatidis* (*Bd*). Chytridiomycosis is lethal to many species of amphibians (Berger et al. 1998, 2005; Lips et al. 2006; Skerratt et al. 2007), and outbreaks of chytridiomycosis have dramatically reduced the species richness, abundance, and population sizes of amphibians globally (Berger et al. 1998; Lips et al. 2006; Schloegel et al. 2006; Skerratt et al. 2007; Crawford et al. 2010). Genetic studies suggest that *Bd* is a recently emerged pathogen that has spread around the world rapidly (James et al. 2009; Farrer et al. 2011), on all continents where amphibians occur.

Chytridiomycosis outbreaks leading to the rapid extirpation of some species of amphibians have occurred across a wide spectrum of climatic regions. For example, the disease has devastated species in deserts in temperate North America (e.g., *Rana yavapaiensis* and *Rana chiricahuensis;* Bradley et al. 2002), in alpine regions in North America (e.g., *Rana muscosa*; Briggs et al. 2010; Vredenburg et al. 2010), and in rainforests in tropical Central America (e.g., *Craugastor punctariolis*; Ryan et al. 2008) and Australia (e.g., *Taudactylus acutirostrostris*, Schloegel et al. 2006). Although not all species (or even all populations) in these climatic regions have experienced mass-mortality events or significant declines, some have been dramatically impacted (Schloegel et al. 2006; Crawford et al. 2010). Additionally, in populations that survive initial outbreaks, recovery to pre-decline levels seems to be slow, possibly due to low survival rates in *Bd*-infected amphibian populations (Rachowicz et al. 2006; Murray et al. 2009).

Temperature is thought to be an important factor in the epidemiology of chytridiomycosis for several reasons. Generally, temperature is known to be a key factor regulating amphibian immune responses (Wright and Cooper 1981; Zapata et al. 1992). More specifically for chytridiomycosis, many field studies (though not all) have found that disease is associated with changes in environmental temperatures (Berger et al. 2004; Woodhams and Alford 2005; Bosch et al. 2007). In some tropical regions, for example, *Bd* outbreaks occur in upland sites where temperatures are cool (Berger et al. 2004; Lips et al. 2006) and seasonal low temperatures are significantly related to high *Bd* prevalence in areas where *Bd* is endemic (Woodhams and Alford 2005). The link between disease and temperature is further supported by laboratory experiments. Temperature has strong effects on *Bd* growth; optimal growth of *Bd* in culture occurs within a temperature range of approximately 17–25°C (Piotrowski et al. 2004; Woodhams et al. 2008). Additionally, experimentally inoculated frogs show clinical signs of infection and die within this optimal temperature window, but are “cured” in higher temperature treatments (>25°C; Woodhams et al. 2003; Chatfield and Richards-Zawacki 2011). Although the mechanisms of thermal effects on both amphibian immunity and *Bd* growth are still the subject of investigation, it is widely thought that temperature is a key factor for disease development at least on the higher end of *Bd*'s thermal optimum (e.g., in tropical regions). It is less clear, however, how thermal effects influence disease dynamics in areas that experience sub-optimal temperatures for *Bd* growth (Knapp et al. 2011).

Given the impact of *Bd* on amphibian populations in multiple climatic zones, and the role that temperature is thought to play in chytridiomycosis outbreaks, many questions regarding the relationship between *Bd* pathogenesis and environmental conditions need to be addressed. Have different strains of *Bd* become differentially adapted to thermal conditions in different climatic regions? Or is *Bd* a “temperature generalist” and pathogenic to amphibians across wide range of temperatures? How does the environment mediate *Bd* virulence once the pathogen is endemic in amphibian populations? And how might disease dynamics shift with global climate change?

Although temperature undoubtedly influences a wide range of factors in chytridiomycosis dynamics, our study specifically addresses how temperature mediates *Bd* reproductive biology. During its asexual reproductive life cycle, motile zoospores colonize amphibian skin and form thalli, which mature into zoosporangia (Longcore et al. 1999; Berger et al. 2005). New zoospores develop within the zoosporangia and are released into the environment to re-infect the individual host or to colonize other hosts nearby (Longcore et al. 1999; Berger et al. 2005). We are interested in the growth of *Bd* and the production of *Bd* zoospores because pathogenesis has been linked with increasing *Bd*-loads in susceptible amphibian hosts; clinical signs of disease and mortality occur in individuals with the highest *Bd*-loads (Voyles et al. 2009; Vredenburg et al. 2010). We know that the generation time and fecundity of *Bd* can be influenced by temperature (Woodhams et al. 2008). It is not known, however, what the long-term responses of *Bd* are to different thermal environments, which may provide insights into *Bd* virulence, disease development, transmission potential, and the likelihood of lethal chytridiomycosis outbreaks.

## Materials and methods

**Figure 1:**
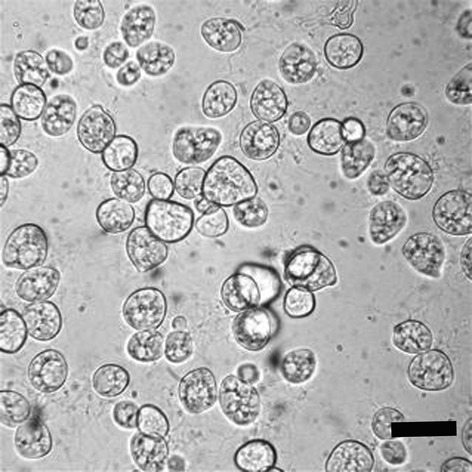
*Batrachochytrium dendrobatidis*. Light micrograph of live sporangia containing zoospores. Scale bar = 10 μm. Isolate Melbourne-L.lesueuri-00-LB-1 was revived from cryo-preservation and two lineages were propagated in two different thermal conditions.

Although other studies have documented short-term effects of temperature on *Bd* (Piotrowski et al. 2004; Woodhams et al. 2008), we investigated *Bd*'s long-term responses to different thermal conditions by integrating empirical laboratory experiments and mathematical modeling approaches. We selected a single *Bd* isolate (Fig 1) and propagated multiple replicates of the isolate in high (23°C) and low (4°C) temperature treatments. The propagated lineages were named for their respective treatments: High Temperature History (HTH) and Low Temperature History (LTH). For each lineage and each experimental temperature treatment, we quantified *Bd* fecundity, the timing of zoospore release, and zoospore activity. We then performed reciprocal temperature transplant experiments, transferring the HTH and LTH lineages to both low and high temperature treatments. Based on our empirical results, we developed a mathematical model of the dynamics of *Bd* in culture, inferred model parameters from data, and used the parameterized model to calculate the intrinsic growth rate of *Bd* in each treatment.

### *Culture of* Batrachochytrium dendrobatidis

The isolate, Melbourne-L.lesueuri-00-LB-1 (Fig 1), was originally obtained from a diseased juvenile *Litoria lesueuri*, cultured on tryptone/gelatin hydrolysate/lactose (TGhL) agar with antibiotics (Longcore et al. 1999), and then cryoarchived (Boyle et al. 2003). We revived aliquots of the cryoarchived culture (Boyle et al. 2003) and passaged *Bd* into liquid TGhL broth (16 g tryptone, 4 g gelatin hydrolysate, 2 g lactose, 1000 mL distilled water; Longcore et al. 1999; Boyle et al. 2003). Two 25-cm^2^ cell culture flasks containing “Low Temperature History” culture (LTH) were incubated at 4°C and passaged every 2–3 months by transferring 2 mL of actively growing culture into 8 mL of new TGhL liquid medium. After 38 months, a second aliquot of the same cryoarchived isolate, Melbourne-L.lesueuri-00-LB-1, was revived and passaged into liquid TGhL broth using identical procedures. These “High Temperature History” cultures (HTH) were maintained at 23°C and passaged every 4–6 days, when zoospore density was near maximum levels (determined by microscopic inspection). When LTH and HTH cultures were matched in passage history (16 passages), a series of plate experiments (reciprocal temperature transplant experiments replicated three times) were conducted.

### Preparation of inocula

LTH and HTH cultures were filtered through a sterile filter paper (Whatman filters, Number 3) to remove sporangia. Cultures were centrifuged (500 g × 10 min), removing the supernatant and re-suspending the zoospores in sterile dilute salt solution (in mMol: KH_2_PO_4_ (1), CaCl_2_.H_2_O (0.2), MgCl_2_.2H_2_O (0.1)). Zoospore concentrations were determined using a hemocytometer (Improved Neubauer Bright-line) and adjusted as needed by addition of dilute salt solution to a concentration of 120 × 10^4^ zoospores per mL. The LTH and HTH zoospore inocula (50 μL) were each pipetted into 25 wells of six sterile 96-well plates. Six plates were organized with two sections (for LTH and HTH inocula) of wells, each containing 50 μL TGhL medium and a perimeter of 36 wells with 100 μL sterile water to avoid evaporation of the wells containing *Bd* inocula. We microscopically examined all wells to ensure that experimental cultures contained only zoospores. Any wells that contained sporangia were excluded from subsequent sampling.

### Reciprocal temperature transplant experiment

Six identically organized plates were created as outlined above, each containing both LTH and HTH cultures. Three plates were incubated at 23°C and three plates at 4°C to allow examination of the responses of the cultures to the two thermal conditions. Plates were inspected daily using light microscopy to monitor zoospore encystment and maturation of the zoosporangia. Once the maturing zoosporangia released the first zoospores, zoospore densities were quantified. We were primarily concerned with quantifying viable zoospores and excluding dead zoospores, which is challenging with photographic images alone. To optimize the quantification of viable (i.e., swimming) zoospores, we randomly selected 10 wells from the LTH and HTH sections of each plate, carefully drew off 30 μL of culture (to avoid collected dead zoospores which accumulate at the bottom of the plate) and counted viable zoospore numbers using a hemocytometer (Voyles 2011). This procedure was repeated daily (10 wells per day) for plates at 23°C because they contained faster growing cultures. The procedure was repeated every 7 days (10 wells per day) for the slower-growing cultures held at 4°C.

### Model development

In order to look at the full shape of the responses in the two lineages and to understand subtle differences between the two, we developed a mathematical model to analyze our empirical data. We fit a delay differential equation model to the data on the concentration of zoospores produced in the next generation from the initial cohort of zoospores placed in each well of the 96-well plate. The model follows the dynamics of: C(t)= the concentration of the initial cohort of zoospores; S(t)= the concentration of zoospore-producing sporangia; and Z(t)= the concentration of zoospores in the next generation. The initial cohort of zoospores, C(t), starts at a concentration of 120 × 10^4^ zoospores per mL, and zoospores in this initial cohort settle and become sporangia at rate *s*_*r*_, or die at rate *μ*. *f*_*s*_ is the fraction of sporangia that survive to the zoospore-producing stage. We assume that it takes a minimum of *T*_*min*_ days before the sporangia produce zoospores, after which they produce zoospores at rate *h*. Zoospore-producing sporangia die at rate *d*_*s*_. The concentration of zoospores, Z(t), is the state variable actually measured in the experiments, and it is assumed that these zoospores settle (*s*_*r*_) or die (*μ*) at the same rates as the initial cohort of zoospores.

The equations that describe this are as follows:


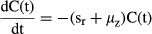



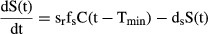



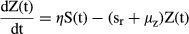


Zoospore-producing sporangia die at rate *d*_*s*_. For this model, the population growth rate *λ* can be calculated numerically from the transcendental equation:


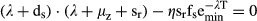


We used a Bayesian model-fitting approach to determine the values of the model parameters in Equations (1)–(3) that best fit the experimental data from each *Bd* lineage grown at each temperature. Our data consist of observations on the numbers of zoospores not from the initial cohort (i.e., on Z(t)), although we know the initial conditions of all the states. Because our observations are in discrete numbers of zoospores, we assume that our observations of the system at set of discrete times t′ are independent Poisson random variables with a mean given by the solution of Equations (1)–(3), at times t′:


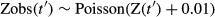


The likelihood of the data given the parameters, underlying model, and initial conditions is then a product over the n observations at each time point in t′. In our analysis, we considered a randomly chosen subset (*n* = 5) of the data at each time point. Uninformative priors were specified for all parameters. The solution of Equations (1)–(3) was not analytically tractable. Thus, as part of the likelihood calculation, the solution to the system of equations was found numerically using a modified version of the PBSd dissolve package in R. We collected 15,000 posterior samples for each experimental case using Markov-chain Monte Carlo (MCMC; Clark 2007).

We confirmed that, within each temperature treatment, the behavior of the *Bd* lineages were significantly different by fitting the model to the data from the two lineages combined (i.e., assuming both lineages have the same parameters) and comparing this to the fit of the model to each lineage separately (i.e., assuming different parameters for the two lineages) using Deviance Information Criterion (DIC).

## Results

### Comparison between 23°C and 4°C

**Figure 2:**
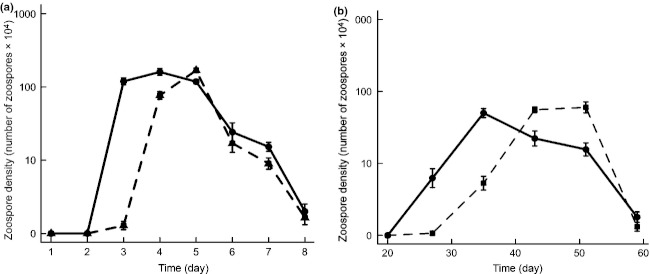
Zoospore densities (mean densities ± SEM) over time in two *Bd* lineages serially propagated at 4°C (“Low temperature history”, LTH lineage; solid lines) and at 23°C (“High temperature history”, HTH lineage; dashed lines). Note the different time scales between A and B.

At 23°C, the *Bd* lifecycle is completed more rapidly than at 4°C. Zoospores from both HTH and LTH lineages encysted more rapidly, produced zoospores more rapidly, released larger numbers of zoospores on the first day of zoospore release, and had shorter periods of zoospore activity in the high temperature treatment than in the low temperature treatment (Fig 2). More specifically, zoospores from both lineages encysted within 24 h at 23°C, but at 4°C zoospores were typically not encysted up to 4 days after plate inoculations. Encysted zoospores of both lineages matured and produced new zoospores in 4 days at 23°C, whereas new zoospores were not observed until 24 days after encystment at 4°C (Fig 2).

**Figure 3:**
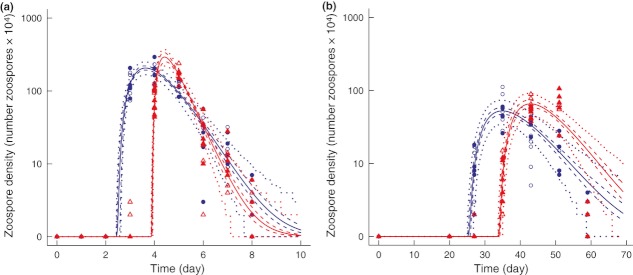
Modeled pathogen population growth rate (lambda, *λ*) at 4°C and 23°C for two lineages of *Batrachochytrium dendrobatidis* (*Bd*)*. Bd* lineages were serially transferred at 4°C (the “Low Temperature History”, LTH lineage; blue, circles) and at 23°C (the “High Temperature History”, HTH lineage; red, triangles). In each panel, lines indicate intrinsic growth rates with 0.95 HPD intervals around the predictions.

### Comparison between HTH and LTH lineages

Although there were similarities between the two lineages across temperature treatments (as described above), the timing of zoospore release and the densities of zoospores on the first day of release differed between lineages within culture temperatures. At 23°C LTH lineages released zoospores on day 3 whereas HTH lineages did not release zoospores until day 4 (Fig 2A). At 4°C LTH lineages released zoospores on day 27 whereas HTH lineages released zoospores on day 35 (Fig 2B).

### *Modeling* of Bd *intrinsic population growth rate under different thermal regimes*

Our model of *Bd* in culture is based on three equations that describe the structure of our experimental data, which include the initial number of zoospores in culture, the abundance of sporangia that can produce zoospores, and the abundance of new zoospores. We generated the intrinsic population growth rate (*λ*) for the *Bd* lineages in each treatment using posterior samples of the model parameters (see methods). Posterior distributions for all parameters are presented in Table 1.

**Table 1:** Model parameters for Bd growth and population growth rate

Parameter	Description	Temp	LTH	HTH
*f*_*s*_	fraction of zoospores that survive	23°C	0.850 (0.571–1.00)	0.935 (0.767–0.998)
		4°C	0.384 (0.182–0.898)	0.241 (0.162–0.439)
*d*_*s*_	sporangia death	23°C	1.36 (1.07–1.84)	1.68 (1.54–1.81)
		4°C	0.174 (0.119–0.247)	0.203 (0.148–0.266)
*η*	rate of zoospore production	23°C	18.4 (11.8–30.0)	38.5 (26.9–51.4)
		4°C	1.83 (0.529–4.52)	3.75 (1.79–7.08)
*T*_*min*_	min. time to zoospore production	23°C	2.49 (2.43–2.57)	3.90 (3.87–3.92)
		4°C	25.4 (25.1–25.8)	34.0 (33.7–34.2)
*s*_*r*_	settle rate	23°C	1.34 (0.840–1.76)	4.72 (3.00–5.96)
		4°C	0.0968 (0.0420–0.182)	0.107 (0.0410–0.179)
*μ*	rate of zoospore death	23°C	0.630 (0.274–0.995)	1.15 (0.504–2.17)
		4°C	0.136 (0.0528–0.193)	0.125 (0.0619–0.193)
*λ*	population growth rate	23°C	0.558 (0.505–0.607)	0.607 (0.547–0.659)
		4°C	0.0161 (0.00949–0.0240)	0.0170 (0.0127–0.0214)

Parameters for a model of *Bd* growth and the intrinsic population growth rate (lambda, *λ*) for two lineages of *Bd* propagated at 4°C (Low Temperature History, LTH) and 23°C (High Temperature History, HTH). Values (medians with highest probability density intervals, HPD = 0.95) reflect lineage growth through time at 4°C and 23°C.

Regardless of the temperature history, *Bd* had lower population growth rates (lambda, *λ*), but longer periods of zoospore activity (settle rate, *s*_*r*,_ and mortality rate, *μ*) in the low temperature treatment (4°C) compared to the high temperature treatment (23°C). For both lineages, *Bd* had a higher intrinsic population growth rate at 23°C (LTH: *λ* = 0.558; HTH: *λ* = 0.607) than at 4°C (LTH *λ* = 0.0161; HTH *λ* = 0.0170). All other parameters showed marked differences between the two thermal treatments (Table 1), suggesting that all parameters contributed to the net effects on population growth rate. Of particular interest are zoospore settle rates (*s*_*r*_) as well as the zoospore mortality rates (*μ*), which indicate the duration of zoospore activity in the two temperature treatments. The 95% credible intervals of the posterior distribution of *s*_*r*_ for both lineages in the high temperature treatment are higher (and have no overlap with) the posterior distribution of *s*_*r*_ in the low temperature experiment (Table 1). The same is true for *μ* (Table 1).

Although the two lineages had relatively similar population growth rates within each thermal treatment, some of the parameters indicate consistent differences between LTH and HTH lineages in both temperature conditions (Table 1). For example, both the rate of zoospore production (*η*) and the minimum time to zoospore production (*T*_*min*_) were lower for the LTH lineage compared to the HTH lineage at both 4°C and 23°C (Table 1).

## Discussion

To investigate the long-term responses of *Bd* to different temperature conditions, we used in vitro serial transfer experiments and quantified changes in *Bd* growth and development. We were particularly interested in temperature-mediated shifts in the *Bd* reproductive life cycle because the density of *Bd* on an amphibian host is associated with the development of lethal chytridiomycosis (Voyles et al. 2009; Vredenburg et al. 2010). We found that *Bd* had lower population growth rates, but longer periods of zoospore activity, in the low temperature treatment (4°C) compared to the high temperature treatment (23°C). We also found that this effect was more pronounced in LTH lineage, but that this outcome did not translate into a population growth rate advantage in lower or higher temperature conditions. These new findings suggest how *Bd* could still be effective at sub-optimal temperatures, even when *Bd* population growth rates are comparatively low.

We propose that *Bd* may be highly effective in low (sub-optimal) thermal conditions because temperature-mediated adjustments that allow zoospores to be active for long periods of time could result in high zoospore encounter rates in amphibian populations. The zoospore encounter rate (i.e., the rate at which an amphibian will encounter a zoospore) is based on the quantity of *Bd* zoospores in the zoospore pool (i.e., on amphibians or in a body of water that contains amphibians; Briggs et al. 2010; Johnson and Briggs 2011). A principal determinant of encounter rates is zoospore production in an individual host (Briggs et al. 2010; Johnson and Briggs 2011). However, encounter rates may also be maintained at high levels if *Bd* zoospores extend their period of viability and reach higher densities in infected individuals or within a zoospore pool. Our study shows that at 4°C, which is below *Bd*'s thermal optima (Piotrowski et al. 2004; Woodhams et al. 2008), *Bd* had lower population growth rates, but longer periods of zoospore activity, compared to the high temperature treatment (23°C). The longer period of activity is characterized by lower zoospore settlement rates (*s*_*r*_), lower zoospore mortality rates (*μ*), and lower sporangia death rates (*d*_*s*_). If this outcome is consistent for *Bd* in nature, then zoospore encounter rates are probably altered in low temperatures. Although this effect remains to be tested in wild amphibian populations, the shifts in *Bd* reproductive cycle that we observed provide new insights into the mechanisms by which *Bd* can be effective across a wide variety of temperature conditions, even below *Bd*'s thermal optimum.

Our results also suggest that *Bd* may respond to selection imposed by different thermal regimes over multiple generations. Using reciprocal temperature transplant experiments, we found that the changes in the *Bd* reproductive life cycle were more pronounced in the LTH lineage. Intrinsic rates of population increase were similar between the two lineages, but zoospores were active significantly longer in the LTH lineage. For example, the minimum time to zoospore production (*T*_*min*_) was consistently shorter for the LTH lineage in both thermal treatments. This result suggests that the LTH lineage may have had a shift in its response to lower temperatures, culminating in longer periods of zoospore activity irrespective of the thermal conditions. These results should be interpreted with caution, however, because the time that these lineages were maintained in artificial media is a confounding variable. It is currently unknown how laboratory maintenance might alter *Bd* isolates, but if these results do reflect adaptive changes, then these adjustments occurred relatively quickly, after a small number (16) of passages.

Previous studies have shown that *Bd*'s growth rate is mediated by temperature (Piotrowski et al. 2004; Woodhams et al. 2008), but our experimental approach and model development differ in important ways. Woodhams et al. (2008) found that more zoospores were produced at 10°C compared to 23°C, but the modeled population growth rate was lower at 10°C compared to 23°C, presumably because the increase in *Bd* fecundity was not sufficient to offset time to zoospore production. One important consideration, however, is that the Woodhams model assumed that *Bd* zoosporangia produce zoospores at a fixed time point following encystment (Woodhams et al. 2008). Our experimental approach allowed us to quantify zoospore production for multiple days post encystment. We found that a model that includes both a minimum number of days until the start of zoospore production (*T*_*min*_) and a continued rate of zoospore production (*η*) better captured net population growth.

Overall, our findings suggest that temperature may dramatically alter multiple parameters for chytridiomycosis dynamics, including the rates of addition to the zoospore pool, loss from the zoospore pool, successful zoospore transmission, and therefore the zoospore encounter rates. Zoospore encounter rates are particularly important because previous models have indicated that frog populations can be rapidly driven to extinction when encounter rates are high (Briggs et al. 2010; Johnson and Briggs 2011). We suggest that this scenario could occur in low (sub-optimal) temperatures if zoospores remain viable for longer periods of time. Additionally, adaptive adjustments in the *Bd* reproduction life cycle that similarly result in prolonged periods of zoospore activity could also transform disease dynamics for frog populations. Thus, the environment and its direct effects on *Bd* could alter the propensity for *Bd* to cause frog population collapse and warrant further investigation.

The adaptive response of *Bd* to temperature is one, but not the only, factor that should be considered as researchers seek to understand the determinants of *Bd* pathogenicity and evolution of virulence. As *Bd* spreads into new hosts and locations, adaptive changes are likely to occur as it becomes endemic. However, host defenses against *Bd*, including host immune responses, body condition, and host behavioral selection of thermal environments, will probably change as well. Even in a relatively simple system (e.g., with only a single susceptible host species), the complex interactions between amphibian hosts and *Bd* should co-evolve and will be influenced by the hosts' and pathogens' shared environmental conditions.

Understanding the temperature-related effects and potentially the adaptive responses of *Bd* will be especially important in the context of global climate change. The “chytrid-thermal-optimum hypothesis” (Pounds et al. 2006) suggests that global climate change might drive chytridiomycosis outbreaks, causing amphibian extinction events. Under this hypothesis, amphibian habitats that become more homoeothermic, due to daytime cooling and nighttime warming, will accelerate disease development by creating a thermal optimum for *Bd* proliferation (Pounds et al. 2006). This hypothesis is highly controversial (Alford et al. 2007; Lips et al. 2008; Rohr et al. 2008) and largely untested. The results of the present study, which are based on experimental results and mathematical modeling, highlight the importance of understanding *Bd* biology at the interface of host, pathogen and environment, and suggest that the effect of temperature on this disease system is likely to be far more complex than initially suspected.
